# Longitudinal minimal residual disease assessment in multiple myeloma patients in complete remission – results from the NMSG flow-MRD substudy within the EMN02/HO95 MM trial

**DOI:** 10.1186/s12885-022-09184-1

**Published:** 2022-02-05

**Authors:** Alexander Schmitz, Rasmus Froberg Brøndum, Hans Erik Johnsen, Ulf-Henrik Mellqvist, Anders Waage, Peter Gimsing, Davine Hofste op Bruinink, Vincent van der Velden, Bronno van der Holt, Markus Hansson, Niels Frost Andersen, Ulf Christian Frølund, Carsten Helleberg, Fredrik H. Schjesvold, Lucia Ahlberg, Nina Gulbrandsen, Bjorn Andreasson, Birgitta Lauri, Einar Haukas, Julie Støve Bødker, Anne Stidsholt Roug, Martin Bøgsted, Marianne T. Severinsen, Henrik Gregersen, Niels Abildgaard, Pieter Sonneveld, Karen Dybkær

**Affiliations:** 1 Department of Haematology, Aalborg University Hospital, Forskningens Hus, Søndre Skovvej 15, DK-9000 Aalborg, Denmark; 2 Clinical Cancer Research Center, Aalborg University Hospital, Aalborg, Denmark; 3 Department of Clinical Medicine, Aalborg University, Aalborg, Denmark; 4 For the Nordic Myeloma Study Group (NMSG) , https://www.nordicmyeloma.org/; 5 South Elvsborg Hospital, Boras, Sweden; 6 Norwegian University of Science and Technology and St. Olav’s University Hospital, Trondheim, Norway; 7 Department of Haematology, University of Copenhagen, Copenhagen, Denmark; 8 Department of Immunology, Erasmus MC, University Medical Center, Rotterdam, The Netherlands; 9 HOVON Data Center, Department of Haematology, Erasmus MC Cancer Institute, Rotterdam, The Netherlands; 10 Skane University Hospital, Lund, Sweden; 11 Department of Haematology, Aarhus University Hospital, Aarhus, Denmark; 12 Department of Haematology, Sjællands Universitetshospital, Roskilde, Denmark; 13 Department of Hematology, Herlev Hospital, Herlev, Denmark; 14 Oslo Myeloma Center, Department of Haematology, Oslo University Hospital, Olso, Norway; 15 KG Jebsen Center for B Cell Malignancies, University of Oslo, Oslo, Norway; 16 Division of Hematology, Linkoping University Hospital, Linkoping, Sweden; 17 NU Hospital Group, Uddevalla hospital, Uddevalla, Sweden; 18 Sunderby Hospital, Luleaa, Sweden; 19 Department of Haematology, Stavanger University Hospital, Stavanger, Norway; 20 Department of Haematology, Odense University Hospital, Odense, Denmark; 21 For the European Myeloma Network (EMN) , https://www.myeloma-europe.org/

**Keywords:** Multiple myeloma, Minimal residual disease, Flow cytometry

## Abstract

**Background:**

Multiple myeloma remains an incurable disease with multiple relapses due to residual myeloma cells in the bone marrow of patients after therapy. Presence of small number of cancer cells in the body after cancer treatment, called minimal residual disease, has been shown to be prognostic for progression-free and overall survival. However, for multiple myeloma, it is unclear whether patients attaining minimal residual disease negativity may be candidates for treatment discontinuation. We investigated, if longitudinal flow cytometry-based monitoring of minimal residual disease (flow-MRD) may predict disease progression earlier and with higher sensitivity compared to biochemical assessments.

**Methods:**

Patients from the Nordic countries with newly diagnosed multiple myeloma enrolled in the European-Myeloma-Network-02/Hovon-95 (EMN02/HO95) trial and undergoing bone marrow aspiration confirmation of complete response, were eligible for this Nordic Myeloma Study Group (NMSG) substudy. Longitdudinal flow-MRD assessment of bone marrow samples was performed to identify and enumerate residual malignant plasma cells until observed clinical progression.

**Results:**

Minimal residual disease dynamics were compared to biochemically assessed changes in serum free light chain and M-component. Among 20 patients, reaching complete response or stringent complete response during the observation period, and with ≥3 sequential flow-MRD assessments analysed over time, increasing levels of minimal residual disease in the bone marrow were observed in six cases, preceding biochemically assessed disease and clinical progression by 5.5 months and 12.6 months (mean values), respectively. Mean malignant plasma cells doubling time for the six patients was 1.8 months (95% CI, 1.4–2.3 months). Minimal malignant plasma cells detection limit was 4 × 10–5.

**Conclusions:**

Flow-MRD is a sensitive method for longitudinal monitoring of minimal residual disease dynamics in multiple myeloma patients in complete response. Increasing minimal residual disease levels precedes biochemically assessed changes and is an early indicator of subsequent clinical progression.

**Trial registration:**

NCT01208766

**Supplementary Information:**

The online version contains supplementary material available at 10.1186/s12885-022-09184-1.

## Introduction

Multiple myeloma (MM) is a common malignant gammopathy and associated with a wide spectrum of symptoms [1]. Patients typically present with proliferation of abnormal monoclonal plasma cells (PC) in the bone marrow (BM) and monoclonal protein (M-component) in the serum and/or urine [2]. Therapeutic advancements like autologous stem cell transplant, proteasome inhibitors, immune-modulators and monoclonal antibodies have resulted in significant improvements in treatment response depth and clinical outcome [3]. However, the majority of patients will eventually develop refractory disease, making MM a remaining incurable disease [4].

Precise assessment of response to therapy is of high importance in every phase of MM and consensus recommendations have been developed by the International Myeloma Working Group for uniform reporting of response levels [5]. Complete response is biochemically defined as non-detectable M-component in the serum and urine by immunofixation plus disappearance of any soft tissue plasmacytoma, and detection of < 5% plasma cells in BM. Stringent CR (sCR) includes all CR criteria plus a normal ratio of serum free light chain ratio (sFLCr), together with the absence of monoclonal PCs by immunohistochemistry after counting ≥100 BM PC or immunofluorescence at a sensitivity level of 10^− 2^ [6, 7]. Achievement of a normal sFLC ratio is associated with improved outcomes including overall survival [8, 9].

Relapse is a consequence of the expansion of residual MM cells [5]. These residual MM cells in patients may be detectable as “minimal residual disease” (MRD, also referred to as “measurable residual disease”) by current Next Generation Flow (NGF) or VDJ sequencing methods with which a minimal sensitivity level of 10^− 5^ or 10^− 6^ can be reached [10, 11]. MRD negativity at the 10^− 5^ level or even more informative at the 10^− 6^ level is a strong prognostic factor of progression-free survival and is under consideration as a surrogate trial endpoint to improve the identification of effective treatments [12–15]. Additionally, IMWG guidelines have introduced the term sustained MRD negativity for individuals with MRD negativity confirmed 1 year apart [5].

Multiparametric flow cytometry (MFC) is a widely applicable technique, allowing identification and discrimination between normal and abnormal PCs based on aberrant patterns of protein expression by neoplastic PCs. MFC has been reported to hold diagnostic, prognostic and predictive information and plays an increasingly important role in diagnostics and prognostics of plasma cell disorders [16, 17]. Flow-MRD assessment, the evaluation of absence of phenotypically aberrant PCs using MFC, currently reaches method sensitivities between 10^− 4^ and 10^− 6^ [11].

Treatment of other haematological malignancies, such as acute lymphoblastic leukaemia and chronic myeloid leukaemia, often are guided by MRD results, and achievement of MRD negativity has been endorsed as a purpose the treatment in acute myeloid leukemia [18–20]. However, standardization of MRD testing in MM as well as defining the role of MRD status in guiding treatment decisions remains unanswered and is ongoing [21]. We hypothesized that longitudinal BM flow-MRD measurements would reveal information on depth and dynamics of MRD that potentially could drive treatment decisions and tailor therapeutic strategies in the future. Few studies have longitudinally evaluated MRD in MM [22–24]. Therefore, we aimed to investigate the impact of long-term sequential BM flow-MRD assessments to determine the potential of flow-MRD monitoring in comparison to standard disease monitoring by M-component and sFLC ratio.

## Methods

Study Design: The NMSG MRD substudy was part of the EMN02/HO95 trial (NCT01208766) and enclosed 23 sites from the Nordic countries [25]. Patients were included in the study from March 1, 2012 to March 31, 2014. Patients undergoing BM aspiration for response assessment prior to start of maintenance treatment were eligible for the MRD study [26]. The study was approved by the national and regional committees (see supplementary information for detailed list) on health research ethics and performed in accordance with the good clinical practice regulations and the declaration of Helsinki principles with written informed consent from all included patients.

Specimens and clinical data: Heparin-stabilized BM samples were received (at the NMSG central Biobank at Aalborg University Hospital) for MFC analysis at diagnosis and were, when eligible for the MRD-study, subsequently for molecular response assessment by flow cytometry (1st flow-RA) and following flow-MRD analyses (Table S1). The EMN Data Center (Torino/Italy) had collected data on clinical characteristics, namely dates for inclusion (INCL), clinical response status (RS) and 1st progression, as well as biochemical analysis data including serum protein electrophoresis (SPEP), serum immunofixation (IF) status, and sFLC ratio. Presence of an M protein on SPEP or IF were considered as positive; sFLC ratios outside the 0.26–1.65 range as abnormal [7]. Clinical progression and RS were documented from the participating sites and defined according to IMWG criteria [27, 28]. Reasons for end of follow-up was death, emigration, patients withdrawal from study participation (off-protocol), or October 31st, 2018 (cut-off) for the flow cytometric data. Cut-off for the clinical data was set at September 18th, 2019. Clinical progression and overall survival status were matched to censoring date for data comparability with the flow-MRD data.

Flow-MRD assessment: A modified 1st generation Euroflow antibody 2 tube panel set for PC disorders (PCD) was applied to freshly stained BM aliquots [29]. For more detailed information see supplementary information (supplementary methods and Table S2; Fig. S1) and Op Bruinink et al. 2020 [30–35]. Flow-MRD positivity was defined as the enumeration of ≥40 mPC cells among ≥1 × 10^6^ total nucleated cells across all concatemerized tubes, reflecting a minimum detection limit of 4 × 10^− 5^ (0.004%). Samples with insufficient quality or technical issues were excluded (“inadequate sample”) as outlined in Table 1. Critical parameters for exclusion were total cellularity (< 1 × 10^5^ cells for diagnostic samples; < 1 × 10^6^ cells for MRD samples), aggregation, coagulation, haemodilution, absence of a clear defined mPC population (< 0.1% for diagnostic samples), absence of a nPC population (for MRD samples, < 100 cells), inadequate specimen processing and/or technical issues. Only samples analysed within 52 h after sampling were included into the MRD-analysis, as recommended from current literature [36, 37].

**Table 1:** Flow-MRD response assessment after treatment of multiple myeloma

		Comment	Patients				Pts. with clinical progression (*1)
**(A)**	**Patients registered/included into the study**	**Patients included into the study (Inclusion period: 01-03-2012 - 31-03-2014)**	**136**				**79**
		**Patients with only diagnostic BM sample received (until 31-10-2018)**		**83**			
		**Patients with BM samples for Flow-MRD analysis received (until 31-08-2018)**		**53**			**29**
**(B)**	**1st flow RA (Flow-MRD analysis FU1)**	**Patients with BM samples received for 1st Flow-RA**		**53**			
		**Excluded patients not reaching RS of CR at 1st response assessment**			**31**		**13**
		**Excluded patients (OFF-protocol)**			**1**		**1**
		**Included patients with RS of CR/SCR at 1st response assessment**			**21**		**14**
		**Included with adequate BM sample**				**15**	
		**Excluded with inadequate BM sample**				**6**	
**(C)**	**Longitudinal flow-MRD analysis (FU1-FUX)**	**Patients with BM samples received for longitudinal analysis**		**53**			
		**Best reached RS during the patients course: <VGPR**				**2**	**1**
		**Best reached RS during the patients course: VGPR**				**11**	**10**
		**Best reached RS during the patients course: CR**				**12**	**5**
		**Best reached RS during the patients course: sCR**				**28**	**13**
		**Excluded patients (Off-protocol)**			**2**		
		**Excluded patients not reaching CR over the entire patients course**			**13**		
		**Excluded with inadequate BM samples (until progression)**			**4**		
		**Included patients with reached RS CR/SCR over the patients course**			**34**		
		**Included with ≥ 3 Flow-MRD samples analysed (incl. FU1)**				**20**	**7**
		**Excluded with ≤ 2 Flow-MRD samples analysed (incl. FU1)**				**14**	**7**

Abbreviations: *PR* Partial response, *VGPR* Very good PR, *CR* Complete response, *SCR* Stringent CR, *Flow-MRD* MRD assessment using MFC, *RA* Response assessment, *RS* Clinical response category, *FU* Follow up (FU1 = 1st RA; FUX = subsequent follow ups), *Pts* Patients. Grey Boxes indicate patients included into the final analysis. (*1): at censoring date

Statistical data analysis: A doubling time for mPC expansion in patients with subsequent biochemical and clinical progression was derived by fitting a log-linear model of mPC versus time and dividing log(2) with the regression coefficient. Differences in proportions were tested using Fishers exact test.

## Results

### Detection of mPCs in BM samples from patients in CR/SCR at 1st flow-response assessment

**Fig. 1:**
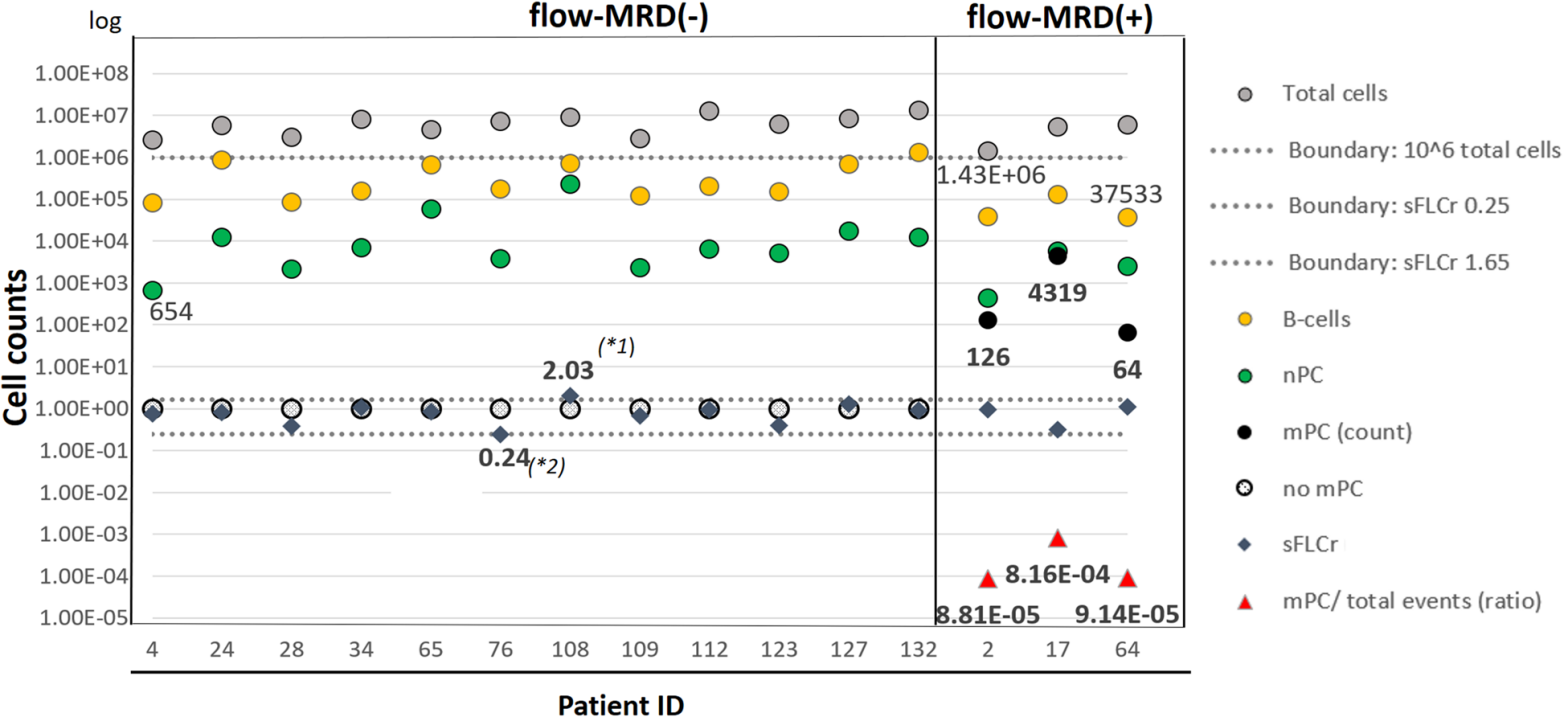
1st Flow-RA of bone marrow sample received from patients at response evaluation. X-axis: Patients with response status of CR and sCR are aligned according to PID number and flow-MRD status. Y-axis: Cell Counts/sFLCr values (log scale). (*1): sFLCr was close to normal range (0.24) and became normal with the next sFLCr measurement (1.58). (*2): sFLCr was taken 27 days after MFC date, previous sFLCr measurement (> 27 days before MFC date) displayed normal ratio. Abbreviations: CR: Complete response. SCR: Stringent complete response. Patient ID / PID: Patient identification number. SFLCr: Serum free light chain ratio. MFC: Multiparamteric flow cytometry. mPC: Malignant plasma cells. nPC: normal polyclonal plasma cells

During the inclusion period, 136 patients (age range 18–64 years) were registered in the NMSG MRD study (Table 1 Part A). In 53 patients, a BM aspirate sample was sent for a first response assessment by flow cytometry (1st flow-RA), when assumed to be in CR/sCR at any time during the course. Fifteen patients with confirmed clinical response status of CR or better (12 with sCR; 3 with CR) and an adequate BM sample were included in the 1st flow-RA analysis (Table 1 Part B; Table S3; Fig. 1). Thirty-eight patients were excluded (31 patients with RS < CR, 1 Off-protocol, 6 with inadequate BM samples). Flow-RA revealed, that 80% (12/15) patients with clinical CR/sCR were mPC negative. Eleven of these patients were in sCR. In the remaining three patients (20%) a small mPC population (64, 126 and 4319, respectively) was detected. Frequency range was 8.2 × 10^− 4^ to 8.8 × 10^− 5^ mPC/ 10^6^ cells. Corresponding sFLC ratios were normal in the three patients (Fig. 1). Time from inclusion to 1st flow-RA varied widely (interval range 2.7–41.6 month, median 20.1 months; lower quartile: 12.6 month; upper quartile: 35.4 months), and sample size varied greatly from 1.3–13.4 × 10^6^ acquired cells (median 5.9 × 10^6^). Due to the small size of the dataset with three flow-RA positive patients, no statistical conclusion could be drawn for rate of clinical progression between the two groups. However, all three flow-RA positive patients subsequently progressed.

### Flow-MRD identifies early progression

Longitudinal flow-MRD assessments were carried out to determine the potential of flow-MRD monitoring on the risk of disease progression and to evaluate its dynamics. The inclusion criterium for this long-term analysis was the achievement of CR during the patient’s course, thereby including patients with VGPR or better at first response assessment. As outlined (Table 1 Part C; Table S3), 34 patients from 53 patients included in the MRD-study fulfilled these criterium, of which 19 patients have been in CR at 1st RA. This group comprises the same 15 patients from the 1st flow-RA analysis plus 4 patients, where 1st flow-RA was not available, but subsequently BM samples for flow-MRD assessment were received. Accordingly, 19 patients (19/53) were excluded (15 patients with best RS of VGPR or lower, 6 patients with only inadequate BM samples, 2 patients went off-protocol).

**Fig. 2:**
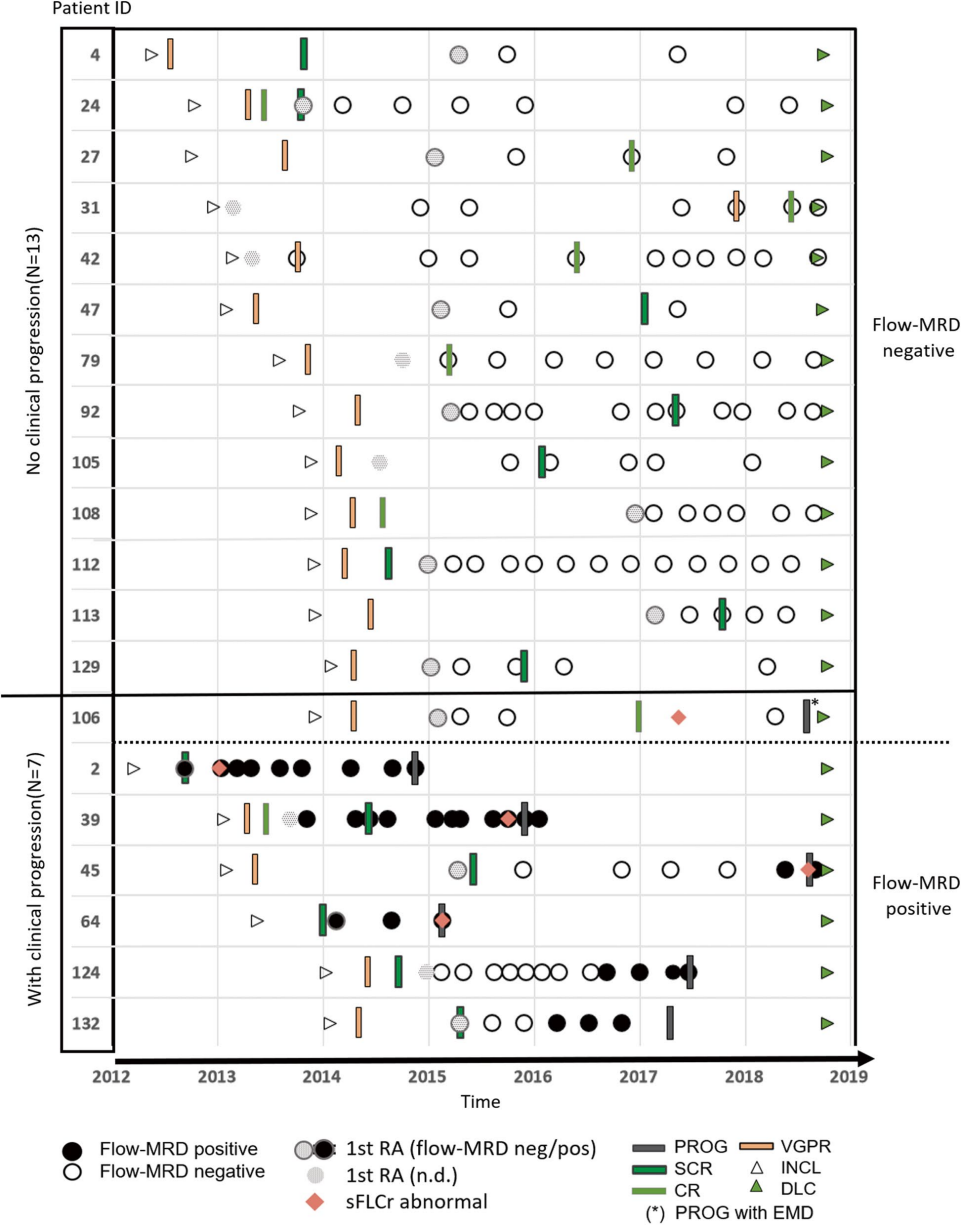
Flow-MRD monitoring of patients reaching CR/sCR during their course, identifies flow-MRD positive patients over time before progression is clinically observed, while flow-MRD negative maintain progression-free regardless their initial RS status. Data points for longitudinal flow-MRD assessment (circles) after inclusion from 20 patients with ≥3 flow-MRD BM samples (inclusive 1st RA), analysed until clinical progression or until date of last clinical contact are lined up over time. Inclusion, RS, progression and date of last contact (until censoring) are indicated. Abbreviations: PR: Partial response. VGPR: Very good PR. CR: Complete response. SCR: stringent CR. INCL: Date of inclusion into the MRD-study. PROG: Date of clinical progression. Flow-MRD: MRD assessment using MFC. RA = Response assessment. RS: Response status (clinical). Patient ID: Patient identification number. SFLCr: Serum free light chain ratio. MFC: Multiparamteric flow cytometry. mPC: Malignant plasma cells. DLC: Date of last contact or censoring date. (*): Patients progressed with extramedullary disease. n.d.: 1st RA BM sample not received or flow-MRD status not determined

For 20 patients (20/34) at least three flow-MRD assessment points with adequate BM samples were analysed (Table 1 Part C; Table S3; Fig. 2). Of these 20 patients, 13 patients displayed sustained flow-MRD negativity upon acquisition of 1.2–19.5 × 10^6^ cells (mean 7.1 × 10^6^; *N* = 105), accompanied by normal values for SFLC ratio, SPEP and IF; and they all remained clinical progression free within the observation period of 6 years (non-progressors). One patient developed extra medullary disease without BM involvement.

**Fig. 3:**
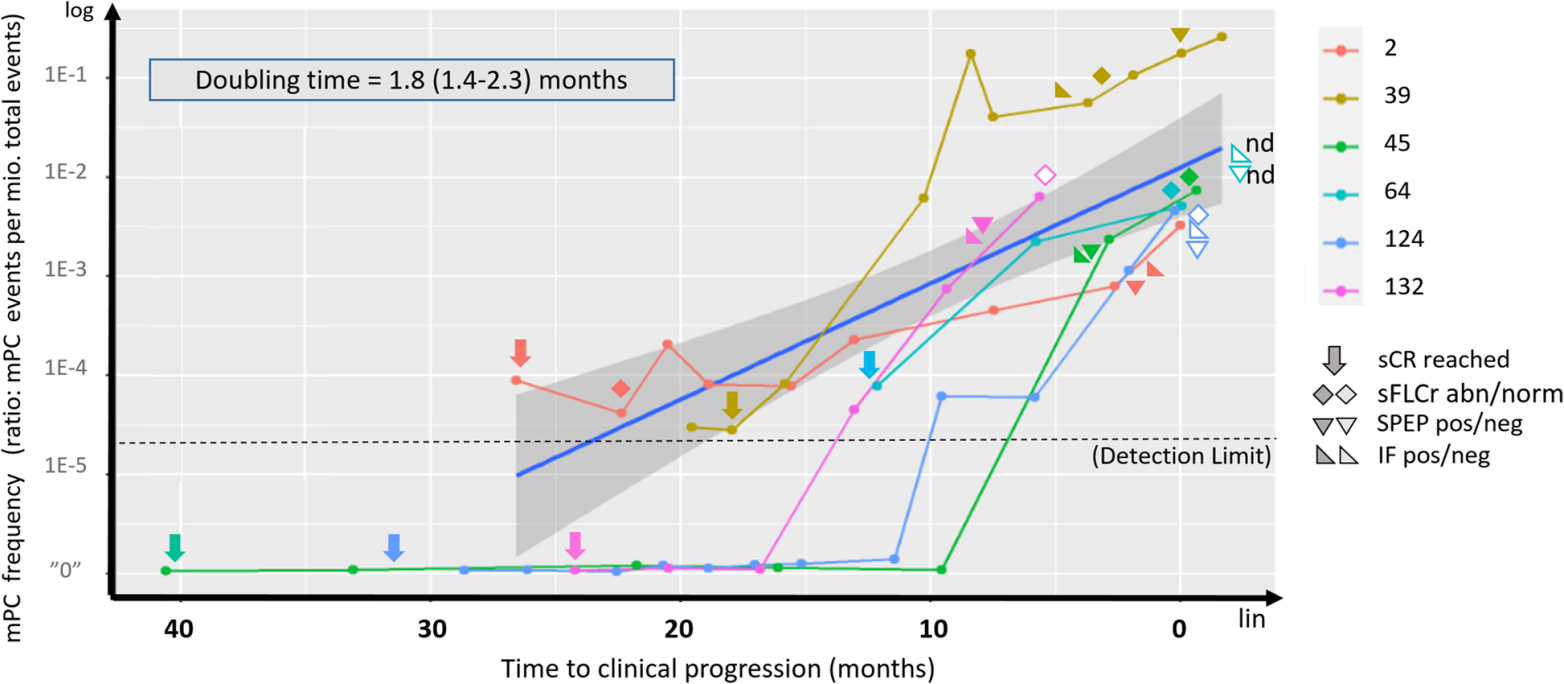
Monitoring the quantity of flow-MRD until clinical progression across six patients with clinical progression reveals, that flow-MRD positivity precedes substantial changes in clinical monitored values for sFLCr, SPEP and IF, and subsequent clinical progression. Time from inclusion to measured flow-MRD positivity varied from 5.9–64.4 month (Mean 19.1 month). MRD positivity was detected with a mean of 12.6 month (range 9.6–26.6) before clinical progression (according to IMWG standard) was recorded. Median mPC doubling time was calculated to 1.8 month (1.4–2.3) across the six patients, fitting a log-linear model for mPC concentration (mPC/ × 10^6^ events) versus time to clinical progression. X-axis: Timeline (months to clinical progression). Y-axis: mPC frequency. Achieved flow-MRD detection limit for the MRD+ patient group is shown as a dotted horizontal line. Abbreviations: MRD: Minimal residual disease. PID: Patient identification ID. SCR: Stringent complete response. SFLCr: Serum free light chain ratio (abnormal/normal). IF: Immunofixation (positive/negative) SPEP: Serum protein electrophoresis (positive/negative). Lin: Linear. Log: Logarithmic. nd: not determined. “0”: Flow-MRD negative

Six patients progressed in the BM (progressors). Longitudinal flow-MRD evaluation revealed flow-MRD positivity followed by mPC population increase for all six progressors upon 3–13 sequential follow-up samples (Median evaluation interval 3.8 months, range 1–30 months; Fig. 3, Table S4). Mean mPC doubling time for the six patients was calculated to be 1.8 months with a confidence interval of 1.4 to 2.3 months, assessed by fitting a log-linear model with the mPC concentration versus time to progression (Fig. 3). Flow-MRD positivity was observed with a median of 12.6 months (IQR 11.8 month, 6 patients) before clinical progression was recorded, and 5.5 months (IQR 9.3, 5 patients) before biochemical parameters became abnormal, which were recurrence of abnormal sFLC ratio in 4 patients and positive SPEP in one patient (Table S2). In patient PID124 all analysed biochemical paramters remained normal until clinical progression.

## Discussion

The primary objective of the flow-MRD study was to evaluate the depth and dynamic of flow-MRD and the risk of disease progression in MM patients in posttreatment CR during lenalidomide maintenance. Our data confirm that flow-MRD is a sensitive tool for response evaluation and subsequent patient monitoring.

First, flow-RA was able to detect residual mPC within the group of patients in CR/sCR, underscoring that flow-RA using the flow-MRD panel has superior sensitivity compared to biochemical and morphological assessment. This observation is in line with previous studies, documenting MRD positivity in MM patients in CR [38, 39]. However, the limited number of patients in the flow-RA positive group did not allow analysis of whether presence of residual mPC at end of treatment evaluation in CR/sCR patients had an impact on progression free survival (PFS) and overall survival (OS) between patients in CR with detectable versus undetectable MRD. These subjects are to be touched upon in upcoming reports for the total EMN02 study [40].

Second, and more important, we determined the prognostic role of MRD by longitudinally monitoring flow-MRD in patients in remission. Focusing on patients with intramedullary disease with achieved CR during the treatment course we showed that MRD appearance, assessed by flow-MRD, predicted biochemical disease progression 5.5 months in advance and 12.6 months in advance to clinical progression. For comparison, in recent studies, where MM patients were sequentially monitored after induction and subsequent autologous stem cell transplantation plus continuous maintenance, a 4 month precedence of MRD positivity towards biochemical relapse and 9 months to clinical relapse were observed [22, 24]. Furthermore, in a cohort of six patients we observed a mean doubling time of less than 2 months (1.8 months with confidence interval 1.4–2.3 months). To the best of our knowledge, no other studies have calculated the dynamics of mPC expansion during pre-clinical relapse. Together, these observations underscore the potential value of sequential flow-MRD assessments. Loss of MRD negativity is the earliest marker of progressive disease and this information could be clinical useful to help clinicians restart treatment before the occururrence of clinical progression and relapse.

IMWG has previously arbitrarily proposed a 12 months interval for monitoring patients with sustained flow-MRD [5]. In our study, based on three cases (PID45, PID124, PID132), the duration from first assessed flow-MRD negativity to flow-MRD positivity was 22.6 months (mean of 11.1, 19.1, 37.7 months). The IMWG proposed 12 months flow-MRD monitoring interval would thus be sufficient for early prediction of progression in 2 out of 3 cases in our study. However, the small number of patients and the wide spread of duration does not allow to draw a meaningful conclusion on a general monitoring interval. Individual patients may differ largely in courses of MRD progression based on risk group, cytogenetic or clonogenc landscape in the malignant cell clones, a larger dataset is needed for robust statistical conclusions [41]. Previously, it was observed, that progressing patients with negative MRD after autologous stem cell transplantation lost their MRD negative status with a median time of 18 months (10 patients), and even shorter intervals of 6 to 12 months for monitoring patients with MRD negative status were suggested [24]. With our observation of a doubling time of 1.8 months for mPC expansion after reaching MRD-positivity, argues for a more frequent flow-MRD monitoring after the initial detection of mPC, to follow their increase in the BM, preceding clinical progression.

Methodologically, the minumum level of flow-MRD detection was defined at study initiation to be 4 × 10^− 5^ (0.004%, ≥40 mPC among ≥10^6^ cells), in order to challenge the IMWG recommendation of 0.01% from 2008. However, consensus criteria for flow-MRD negativity was updated in 2016 to a minimum sensitivity of 10^− 5^, among acquisition of ≥3 × 10^6^ cells [5]. Furthermore, there is now evidence, that patients achieving MRD negativity at the level of 10^− 6^ have prolonged progression-free periods when compared to those who are MRD negative at 10^− 5^ or lower [42, 43]. Although we aimed to analyse 10^6^ cells per BM sample, all available cells from an MRD sample were routinely processed, resulting in 65% of BM samples (across 34 patients) and 86% of BM samples (across 20 patients with ≥3 MRD samples processed) with ≥3 × 10^6^ cells acquired. However, we find, that the acquisition of 1 × 10^6^ events would have been sufficient for the detection and enumeration of MRD-positivity in our patients, and vice versa no clear mPC population was detected in patients with sustained flow-MRD negativity upon aquisition of up to 2 × 10^7^ cells (average 7.0 × 10^6^ across 110 flow-MRD negative BM samples). This suggests that acquisition of 1–3 × 10^6^ events in most cases is sufficient for flow-MRD evaluation, thereby accepting the risk of falsely missing patients with rare residual cells due to sensitivity restrictions in first place in few cases; but those will be detected subsequently when monitored longitudinally.

Achievement of complete response has been accepted as a relevant surrogate marker of survival and attainment of patients attaining sCR has been shown to translate to better survival outcomes compared to conventional CR alone [27, 44]. However, the association between conventional response outcomes and survival in patients with newly diagnosed MM is not clear [45, 46]. Especially, the prognostic value of sFLC measurements in MM patients has been questioned; large studies supporting the role of the assessment and others questioning the role of sFLC in the evaluation of response [47]. In our dataset, flow-MRD negative and non-progressing patients had also normal for sFLC ratios. Among the six progressing patients, we observed changes in the biochemical markers including sFLC ratio in most of the patients (83%), before clinical progression was documented, however, only 50% became sFLC ratio abnormal. These changes were generally detected months later than flow-MRD positivity, underpinning the higher sensitivity of flow-MRD analysis versus biochemical markers, relying on traditional biochemical techniques and plasma cell enumeration by morphology with suboptimal sensitivity. Only PID124 progressed without biochemical abnormality, although flow-MRD detected monoclonal mPC in the BM. It is possible, that this patient has non-secretory malignant cells that may not express and release light chain into the PB at levels detectable with the standard serum measurements. Also measuring M-protein levels are not applicable in patients with oligosecretory or nonsecretory disease, because in these cases the levels of the paraprotein are low or nondetectable [48]. To date, there is no uniform association between rapidity of response and survival outcomes in MM. Studies, but performed before introduction of novel agents, have demonstrated that a rapid decrease of M-protein in the first 1 or 2 cycles of therapy is predictive of longer survival [49]. In a more recent study, slow and gradual response to initial therapy has been shown to be a favourable prognostic factor in MM, with a significantly worse overall survival of early responders compared to late responders [50]. We see the same overall tendency. In the presented study, progressing patients displayed on average a clinical response kinetic with faster CR compared to the non-progressors.

Despite its importance, invasive BM sampling for routine monitoring of MRD has shortcomings by not accounting for spatial heterogeneity and the patchy nature of MM, which may lead to false-negative results. Moreover, undetectable MRD in the BM may hide extramedullary disease, potentially detectable by these technologies [51]. Additionally, it is an invasive procedure [52]. Alternative non-invasive methods for MRD testing, such as analysis of circulating tumour cells or circulating cell-free tumour DNA in liquid biopsies from PB [36, 37]. However, the sensitivity of MRD detection in PB and the optimal method to be used are unclear and further clinical studies are recommended to explore the use of PB for the detection of MRD and for comparison with results obtained in BM, in a similar way as shown for MM at diagnosis [53]. Finally, new sensitive methods, such as quantitative immunoprecipitation mass spectrometry and the MALDI TOF technique may overcome that shortcoming [54, 55].

In conclusion, this study emphasises that flow-MRD assessment is a sensitive and appropriate tool for deeper response evaluation in MM patients beyond CR, and for longitudinal monitoring of MRD with potential detection of early clonal expansion several months prior to biochemical and clinical progression. This offers the opportunity for early intervention and such strategies should be tested in clinical trials.

## Supplementary Information

**Additional file 1.**

## Data Availability

All data generated or analysed during this study are included in this published article and its supplementary information file**.** All biological material has been used.

## References

[1] Palumbo A, Anderson K (2011). Multiple myeloma. N Engl J Med.

[2] Fonseca R, Bergsagel PL, Drach J, Shaughnessy J, Gutierrez N, Stewart AK (2009). International myeloma working group molecular classification of multiple myeloma: spotlight review. Leukemia.

[3] Kumar SK, Rajkumar SV, Dispenzieri A, Lacy MQ, Hayman SR, Buadi FK (2008). Improved survival in multiple myeloma and the impact of novel therapies. Blood.

[4] Ravi P, Kumar SK, Cerhan JR, Maurer MJ, Dingli D, Ansell SM (2018). Defining cure in multiple myeloma: a comparative study of outcomes of young individuals with myeloma and curable hematologic malignancies. Blood Cancer J.

[5] Kumar S, Paiva B, Anderson KC, Durie B, Landgren O, Moreau P (2016). International myeloma working group consensus criteria for response and minimal residual disease assessment in multiple myeloma. Lancet Oncol.

[6] Dispenzieri A, Kyle R, Merlini G, Miguel JS, Ludwig H, Hajek R (2009). International myeloma working group guidelines for serum-free light chain analysis in multiple myeloma and related disorders. Leukemia..

[7] Rajkumar SV, Dimopoulos MA, Palumbo A, Blade J, Merlini G, Mateos M-VV (2014). International Myeloma Working Group updated criteria for the diagnosis of multiple myeloma. Lancet Oncol.

[8] Kapoor P, Kumar SK, Dispenzieri A, Lacy MQ, Buadi F, Dingli D (2013). Importance of achieving stringent complete response after autologous stem-cell transplantation in multiple myeloma. J Clin Oncol.

[9] Alhaj Moustafa M, Rajkumar SV, Dispenzieri A, Gertz MA, Lacy MQ, Buadi FK (2015). Utility of serum free light chain measurements in multiple myeloma patients not achieving complete response to therapy. Leukemia.

[10] Paiva B, Corchete LA, Vidriales M-B, Puig N, Maiso P, Rodriguez I (2016). Phenotypic and genomic analysis of multiple myeloma minimal residual disease tumor cells: a new model to understand chemoresistance. Blood.

[11] Flores-Montero J, Sanoja-Flores L, Paiva B, Puig N, García-Sánchez O, Böttcher S (2017). Next generation flow for highly sensitive and standardized detection of minimal residual disease in multiple myeloma. Leukemia.

[12] Anderson KC, Auclair D, Kelloff GJ, Sigman CC, Avet-Loiseau H, Farrell AT (2017). The role of minimal residual disease testing in myeloma treatment selection and drug development: current value and future applications. Clin Cancer Res.

[13] Paiva B, Vidriales M-B, Pérez JJ, Mateo G, Montalbán MA, Mateos MV (2009). Multiparameter flow cytometry quantification of bone marrow plasma cells at diagnosis provides more prognostic information than morphological assessment in myeloma patients. Haematologica.

[14] Rawstron AC, Child JA, de Tute RM, Davies FE, Gregory WM, Bell SE (2013). Minimal residual disease assessed by multiparameter flow cytometry in multiple myeloma: impact on outcome in the Medical Research Council Myeloma IX Study. J Clin Oncol.

[15] Munshi NC, Avet-Loiseau H, Anderson KC, Neri P, Paiva B, Samur M (2020). A large meta-analysis establishes the role of MRD negativity in long-term survival outcomes in patients with multiple myeloma. Blood Adv.

[16] Johnsen HE, Bøgsted M, Klausen TW, Gimsing P, Schmitz A, Kjaersgaard E (2010). Multiparametric flow cytometry profiling of neoplastic plasma cells in multiple myeloma. Cytometry B Clin Cytom.

[17] Soh KT, Tario JD, Wallace PK (2017). Diagnosis of plasma cell Dyscrasias and monitoring of Minimal residual disease by multiparametric flow cytometry. Clin Lab Med.

[18] Buccisano F, Maurillo L, Schuurhuis GJ, Del Principe MI, Di Veroli A, Gurnari C (2019). The emerging role of measurable residual disease detection in AML in morphologic remission. Semin Hematol.

[19] Bassan R, Brüggemann M, Radcliffe H-S, Hartfield E, Kreuzbauer G, Wetten S (2019). A systematic literature review and meta-analysis of minimal residual disease as a prognostic indicator in adult B-cell acute lymphoblastic leukemia. Haematologica.

[20] Del Giudice I, Raponi S, Della Starza I, De Propris MS, Cavalli M, De Novi LA (2019). Minimal residual disease in chronic lymphocytic leukemia: a new goal?. Front Oncol.

[21] Munshi NC, Avet-Loiseau H, Rawstron AC, Owen RG, Child JA, Thakurta A (2017). Association of Minimal Residual Disease with Superior Survival Outcomes in patients with multiple myeloma. JAMA Oncol.

[22] Oliva S, Gambella M, Gilestro M, Muccio VE, Gay F, Drandi D (2017). Minimal residual disease after transplantation or lenalidomide-based consolidation in myeloma patients: a prospective analysis. Oncotarget.

[23] Ferrero S, Ladetto M, Drandi D, Cavallo F, Genuardi E, Urbano M (2015). Long-term results of the GIMEMA VEL-03-096 trial in MM patients receiving VTD consolidation after ASCT: MRD kinetics’ impact on survival. Leukemia.

[24] Gu J, Liu J, Chen M, Huang B, Li J (2018). Longitudinal flow cytometry identified “Minimal residual disease” (MRD) evolution patterns for predicting the prognosis of patients with transplant-eligible multiple myeloma. Biol Blood Marrow Transplant.

[25] Cavo M, Gay F, Beksac M, Pantani L, Petrucci MT, Dimopoulos MA (2020). Autologous haematopoietic stem-cell transplantation versus bortezomib–melphalan–prednisone, with or without bortezomib–lenalidomide–dexamethasone consolidation therapy, and lenalidomide maintenance for newly diagnosed multiple myeloma (EMN02/HO95): a mult. Lancet Haematol.

[26] Rajkumar SV, Harousseau JL, Durie B, Anderson KC, Dimopoulos M, Kyle R (2011). Consensus recommendations for the uniform reporting of clinical trials: report of the international myeloma workshop consensus panel 1. Blood..

[27] Durie BGM, Harousseau JL, Miguel JS, Bladé J, Barlogie B, Anderson K (2006). International uniform response criteria for multiple myeloma. Leukemia..

[28] Kyle RA, Rajkumar SV (2009). Criteria for diagnosis, staging, risk stratification and response assessment of multiple myeloma. Leukemia.

[29] Kalina T, Flores-Montero J, van der Velden VHJ, Martin-Ayuso M, Böttcher S, Ritgen M (2012). EuroFlow standardization of flow cytometer instrument settings and immunophenotyping protocols. Leukemia.

[30] Hofste op Bruinink D, Oliva S, Rihova L, van der Holt B, Gilestro M, te Marvelde JG (2016). Flowcytometric Minimal residual disease assessment in the EMN-02/HOVON-95 MM trial: used methods and a comparison of their sensitivity. Blood.

[31] Almeida J, Oreao A, Ocqueteau M, Mateo G, Corral M, Caballero MD (1999). High-sensitive immunophenotyping and DNA ploidy studies for the investigation of minimal residual disease in multiple myeloma. Br J Haematol.

[32] Pérez-Persona E, Vidriales M-B, Mateo G, García-Sanz R, Mateos M-V, de Coca AG (2007). New criteria to identify risk of progression in monoclonal gammopathy of uncertain significance and smoldering multiple myeloma based on multiparameter flow cytometry analysis of bone marrow plasma cells. Blood.

[33] Arroz M, Came N, Lin P, Chen W, Yuan C, Lagoo A (2016). Consensus guidelines on plasma cell myeloma minimal residual disease analysis and reporting. Cytom Part B Clin Cytom.

[34] Jelinek T, Bezdekova R, Zatopkova M, Burgos L, Simicek M, Sevcikova T (2017). Current applications of multiparameter flow cytometry in plasma cell disorders. Blood Cancer J.

[35] Op Bruinink DH, Oliva S, Rihova L, Schmitz A, Gilestro M, Te Marvelde J (2020). Standardization of flow cytometric minimal residual disease assessment in international clinical trials. A feasibility study from the European myeloma network. Haematologica.

[36] Rasche L, Alapat D, Kumar M, Gershner G, McDonald J, Wardell CP (2019). Combination of flow cytometry and functional imaging for monitoring of residual disease in myeloma. Leukemia.

[37] Pugh TJ (2018). Circulating tumour DNA for detecting Minimal residual disease in multiple myeloma. Semin Hematol.

[38] Martinez-Lopez J, Lahuerta JJ, Pepin F, González M, Barrio S, Ayala R (2014). Prognostic value of deep sequencing method for minimal residual disease detection in multiple myeloma. Blood.

[39] Munshi NC, Anderson KC (2013). Minimal residual disease in multiple myeloma. J Clin Oncol.

[40] Oliva S, Hofste op Bruinink D, Řĺhová L, Spada S, van der Holt B, Troia R (2017). Minimal residual disease (MRD) monitoring by multiparameter flow cytometry (MFC) in newly diagnosed transplant eligible multiple myeloma (MM) patients: results from the EMN02/HO95 phase 3 trial. J Clin Oncol.

[41] Schinke C, Hoering A, Wang H, Carlton V, Thanandrarajan S, Deshpande S (2017). The prognostic value of the depth of response in multiple myeloma depends on the time of assessment, risk status and molecular subtype. Haematologica.

[42] Paiva B, Puig N, Cedena M-TT, Rosiñol L, Cordón L, Vidriales M-BB (2019). Measurable residual disease by next-generation flow cytometry in multiple myeloma. J Clin Oncol.

[43] Scott SD, Fletcher M, Whitehouse H, Whitby L, Yuan C, Mazzucchelli S (2019). Assessment of plasma cell myeloma minimal residual disease testing by flow cytometry in an international inter-laboratory study: is it ready for primetime use?. Cytom Part B Clin Cytom.

[44] Gay F, Larocca A, Wijermans P, Cavallo F, Rossi D, Schaafsma R (2011). Complete response correlates with long-term progression-free and overall survival in elderly myeloma treated with novel agents: analysis of 1175 patients. Blood..

[45] Martinez-Lopez J, Paiva B, Lopez-Anglada L, Mateos M-V, Cedena T, Vidriales M-B (2015). Critical analysis of the stringent complete response in multiple myeloma: contribution of sFLC and bone marrow clonality. Blood.

[46] Mainou M, Madenidou A-V, Liakos A, Paschos P, Karagiannis T, Bekiari E (2017). Association between response rates and survival outcomes in patients with newly diagnosed multiple myeloma. A systematic review and meta-regression analysis. Eur J Haematol.

[47] García de Veas Silva JL, Bermudo Guitarte C, Menéndez Valladares P, Rojas Noboa JC, Kestler K, Duro Millán R. Prognostic value of serum free light chains measurements in multiple myeloma patients. Lafrenie RM, PLoS One 2016;11(11):e0166841. Available from: 10.1371/journal.pone.0166841 10.1371/journal.pone.0166841 PMC5125636 27893836

[48] Brioli A, Giles H, Pawlyn C, Campbell JP, Kaiser MF, Melchor L (2014). Serum free immunoglobulin light chain evaluation as a marker of impact from intraclonal heterogeneity on myeloma outcome. Blood.

[49] Schaar CG, Kluin-Nelemans JC, Le Cessie S, Franck PFH, Te Marvelde MC, Wijermans PW (2004). Early response to therapy and survival in multiple myeloma. Br J Haematol.

[50] Yan Y, Mao X, Liu J, Fan H, Du C, Li Z (2019). The impact of response kinetics for multiple myeloma in the era of novel agents. Blood Adv.

[51] Mishima Y, Paiva B, Shi J, Park J, Manier S, Takagi S (2017). The mutational landscape of circulating tumor cells in multiple myeloma Europe PMC funders group. Cell Rep.

[52] Gendron N, Chahabi SZ, Poenou G, Rivet N, Belleville-Rolland T, Lemaire P (2019). Pain assessment and factors influencing pain during bone marrow aspiration: a prospective study. PLoS One.

[53] Sanoja-Flores L, Flores-Montero J, Garcés JJ, Paiva B, Puig N, García-Mateo A (2018). Next generation flow for minimally-invasive blood characterization of MGUS and multiple myeloma at diagnosis based on circulating tumor plasma cells (CTPC). Blood Cancer J.

[54] Puig N, Mateos M-V, Contreras T, Paiva B, Cedena MT, Pérez JJ (2019). Qip-mass spectrometry in high risk smoldering multiple myeloma patients included in the GEM-CESAR trial: comparison with conventional and Minimal residual disease IMWG response assessment. Blood.

[55] Eveillard M, Rustad E, Roshal M, Zhang Y, Ciardiello A, Korde N (2020). Comparison of MALDI-TOF mass spectrometry analysis of peripheral blood and bone marrow-based flow cytometry for tracking measurable residual disease in patients with multiple myeloma. Br J Haematol.

