# Supplementary material for: Longitudinal minimal residual disease assessment in multiple myeloma patients in complete remission – results from the NMSG flow-MRD substudy within the EMN02/HO95 MM trial
Source: BMC Cancer. 2022 Feb 5;22:147. doi: 10.1186/s12885-022-09184-1 (PMC8818194; doi:10.1186/s12885-022-09184-1)
Supplement: Supplementary file 1 — Additional file 1. [file 12885_2022_9184_MOESM1_ESM.docx]

**Title: Longitudinal minimal residual disease assessment in multiple myeloma patients in complete remission – results from the NMSG flow-MRD substudy within the EMN02/HO95 MM trial**

Running head: Longitudinal flow-MRD Assessment in CR MM Patients

**---------------------------------------------------------------------------------------------------------------**

Supplementary Appendix:

**1 SUPPLEMENTARY MEHODS**

National and regional study approval

- Participating sites
- **Table S1**: Participating sites in the NMSG MRD study
- Multiparametric flow-MRD analysis
- Antibody staining for MFC
- **Table S2:** Antibody staining panel
- MFC sample acquisition
- MFC analysis (gating strategy)
- **Figure S1**: General MFC analysis gating strategy of normal and malignant cellular BM populations

**2 SUPPLEMENTARY RESULTS**

- **Table S3:** Flow-MRD assessment of BM: 1st flow-RA and longitudinal monitoring
- **Table S4**: Monitoring the quantity of flow-MRD until progression (6 patients)

**3 SUPPLEMENTARY REFERENCES**

1. **SUPPLEMENTARY MEHODS**

**Study Approval**

The study was approved by the national committees on health research ethics and performed in accordance with the good clinical practice regulations and the declaration of Helsinki principles with written informed consent from all included patients

DK: VEK H-1-2010-071/H-20006109
SE: DNR: 653-10
NO: REK 210/2344

**Participating sites**

The Nordic Myeloma Study Group (NMSG) part of the MRD study enclosed 23 sites from the Nordic countries (Denmark, Norway, Sweden), from which diagnostic BM samples from patients (N=136) eligible for the MRD study, were sent to the NMSG central Biobank at Aalborg University Hospital for flow cytometry based immunophenotyping. Subsequently, 17 sites sent follow-up BM samples from 53 patients for assessment of minimal residual disease using flow cytometry (flow-MRD).

*
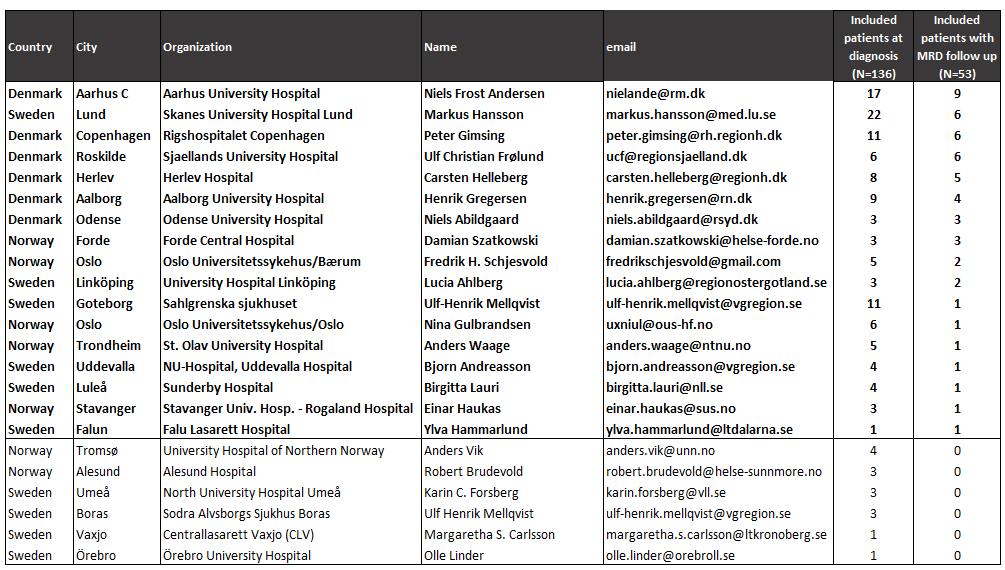
*

***Table S1: Participating NMSG sites and investigators***

**Multiparametric flow cytometry (MFC) for detection and enumeration of diagnostic and flow-MRD follow-up BM samples**

*
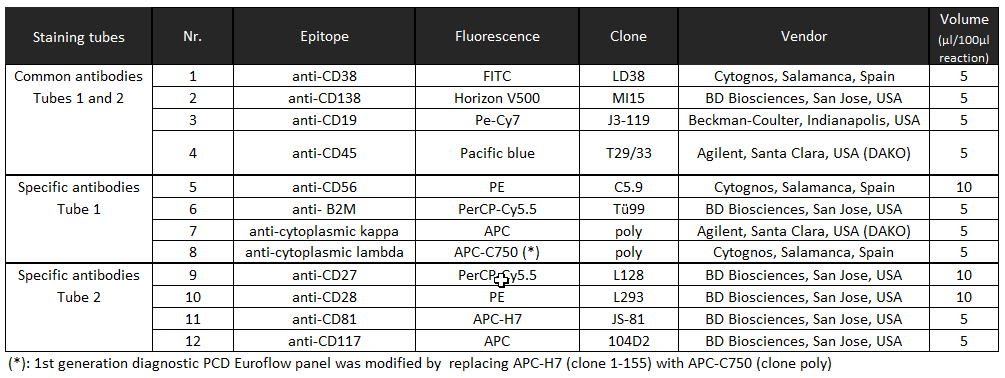
***Antibody staining for MFC:** BM aliquots of 0.1-0.5ml from diagnostic or 1-2ml from follow-up MRD samples were stained using a modified 2 tube diagnostic Euroflow antibody panel set for PC disorders (PCD; Table S2) and processed as published previously by the Euroflow consortium (1). Briefly, samples were counted, washed, resuspended in stain buffer (Stain Buffer BSA, BD Pharmigen) and split equally into two tubes. Tube2 was stained using antibodies 1-4 and 9-12, subjected to red blood cell lysis following manufacturers’ recommendations (Easy Lyse, Beckman Coulter), washed, resuspended in stain buffer and stored until measured in the flow cytometer. Tube 1 was stained first with antibodies 1-6, centrifuged, resuspended and prepared for intracellular staining, using fixation and permeabilization following manufacturers’ recommendations (Caltag, Fix&Perm) and subsequently stained with antibodies 7-8. Incubation time for all staining was 30min at room temperature. Finally, cells were washed, resuspended in stain buffer and stored until measured in the flow cytometer. Since July 2016, all cells were first lysed (“bulk lysis method”) using BulkLysis-solution (Cytognos), resuspended in stain buffer, counted and subsequently stained.(1)

***Table S2: Antibody staining panel for flow-MRD analysis.*** *Common antibodies were included in both staining tubes, specific antibodies in tube 1 or tube 2, respectively. Antibodies 7 and 8 were added after sample fixation and permeabilisation.*

**MFC sample acquisition:** BD FACSCanto^TM^ II and BD FACSAria^TM^ 2 (BD Biosciences) flow cytometers with matched optical 3-laser default configuration were utilized. Cytometers were harmonized, quality controlled after recommendations from Euroflow (1), using CS&T (Becton Dickinson) and 8-peak calibration particles (Spherotech). Spectral overlap compensation settings were generated using compensation capture beads (CompBeads/BD Bioscience or VersaComp/Beckman Coulter) for all used fluorochromes. For diagnostic sample analysis a total of >100.000 cells were acquired, for MRD samples all available cells, at least 1 x 10^6 cells, were analysed by MFC. The sensitivity of the protocol (incl. bulk lysis) has been compared in a quality assessment round, which showed a high correlation of results, compared to three other central MFC MRD laboratories in the Netherlands, Czech Republic and Italy (2). Samples were either acquired ungated and stored as multiple datafiles, or in a 2-step procedure including a boolean “CD19+ OR CD38+” electronic storage-gating after initial acquisition of 50.000-200.000 cellular events without electronic gating (3),(4) Accordingly, the total number of cells for these samples was defined as the number of events without doublets and debris calculated based on the ratio between gated events in the ungated sample versus gated sample using the FlowJo^TM^ (FlowJo, Treestar; USA) software package.

**Gating strategy for enumeration of selected multiple myeloma BM populations from diagnostic and follow-up samples:** Normal and malignant BM populations were defined by expert-based nonautomated data analysis utilizing the Infinicyt software package (Cytognos, Salamanca, Spain); (5),(6). Briefly, flow cytometry data files from acquired tubes were concatemerized without calculation of missing parameters. Debris and doublets were removed based on scatter properties. Malignant plasma cells (mPC) were differentiated from normal plasma cells (nPC) and internal control populations by manual gating using surface expressed markers and the cytoplasmic expression of light chain. Populations were assigned by manually applying gates around the regions of interest using multiple markers simultaneously and removal of events not belonging to the population by simultaneous inspection of relevant marker combinations (*Figure S1*). B-cells were defined by a combination of scatter properties, positive markers (CD45 and CD19), markers with low or absent expression (CD138, CD56, CD28), and further separated based on their CD38 expression into mature B-cells and CD38 positive B-cells. Plasma cells were basically defined by scatter properties and high CD38 expression level together with CD138, CD45 and CD19 expression. Malignant plasma cells were subsequently separated from their normal counterpart based on CD19 negativity and CD45 under expression, in combination with varying levels of CD56, CD27, CD28, CD117, CD81 and clonality (kappa/lambda restriction), whereas normal plasma cells were generally CD56/CD28/CD117low, CD27 positive and polyclonal. Mast cells were defined as bright CD117 positive/CD38 negative. Remaining CD45 positive/side scatter low cells were defined as lymphocyte population.

**Figure S1: General MFC analysis gating strategy for immunophenotypical definition and gating of normal and malignant cellular BM populations**


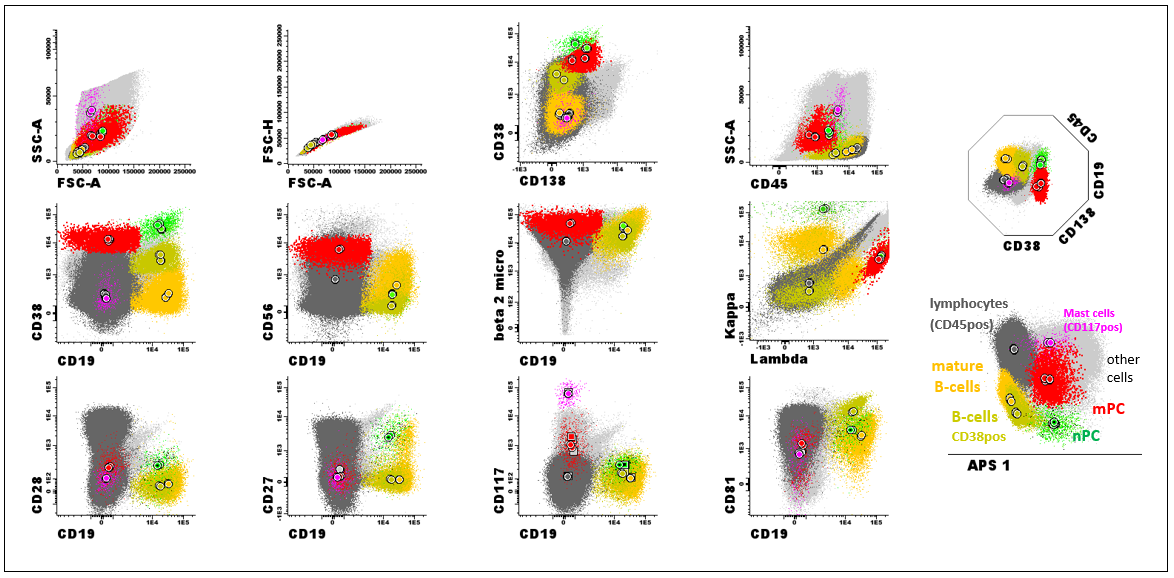


**Figure S1 Legend:** **Assigned populations:** **Red:** mPC (CD38++, CD45 low-dim, CD19low, CD138 variable); **Green:** nPC (CD38++, CD45 dim-pos, CD19pos). **Orange:** mature B-cells CD19pos CD45pos CD38neg). **Olive:** CD38pos B-cells (CD19pos, CD38pos, CD45pos). **Pink:** Mast cells (CD117++; CD38neg). **Dark grey**: other CD45pos lymphocytes. **Light grey:** Unassigned cells. **Circles:** Median MFI values for individual acquired tubes.

**2 SUPPLEMENTARY RESULTS**

**
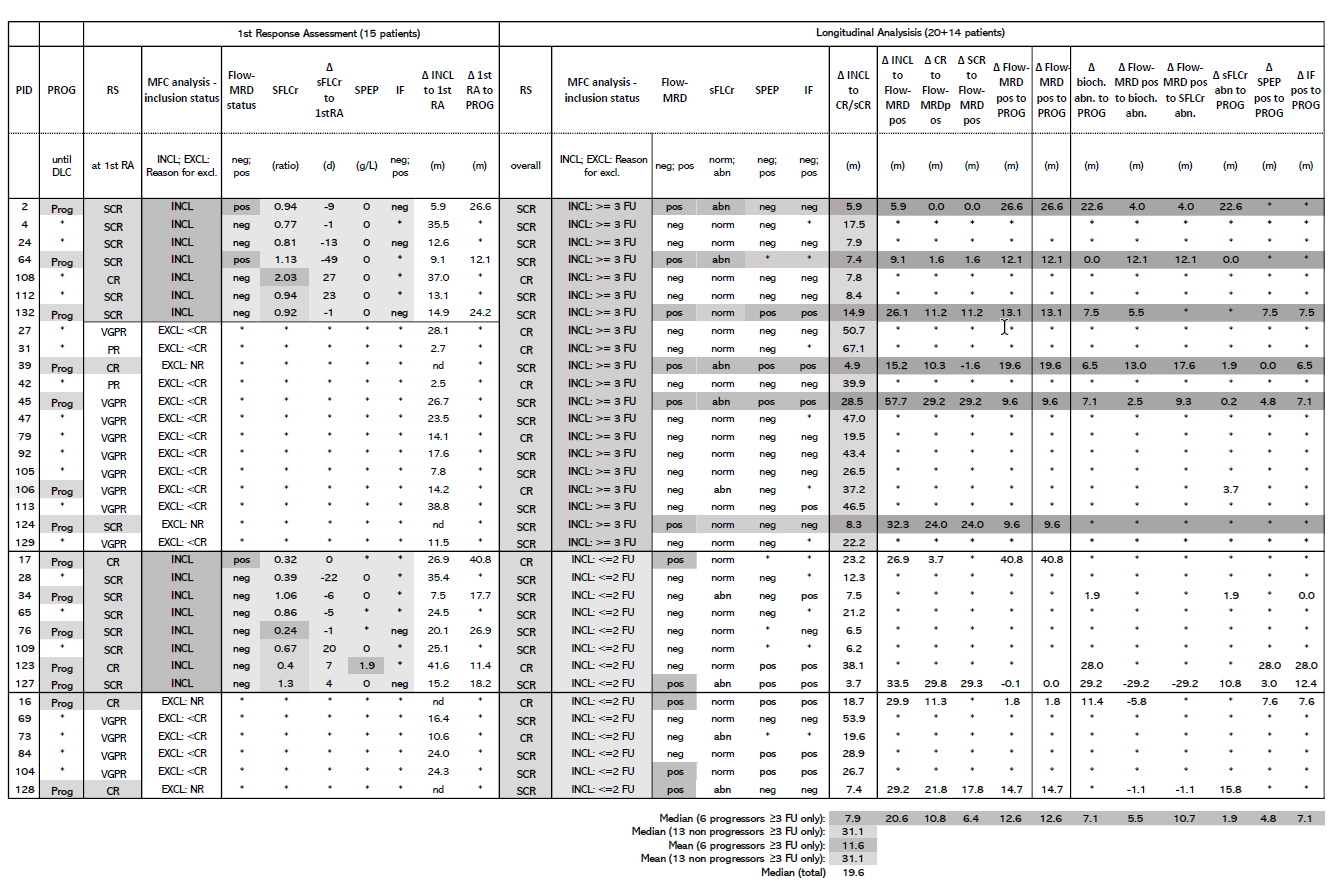
Table S3: Flow-MRD assessment and corresponding clinical data at 1^st^ response assessment (1^st^ RA) and longitudinal monitoring**

**Table S3 Legend:**

From 53 patients included into the MRD-study, 20 patients, achieving a complete response (CR) at any time during the course, were included with longitudinal analysis of ≥3 flow-MRD follow-up samples (**INCL: ≥3 FU**); another 14 patients with two or less FU (**INCL: ≤2 FU**. Patients with overall response of <CR were excluded from the final analysis. Initially, 15 patients, with achieved CR at the time of 1^st^ flow-RA and adequate BM sample, were analysed for the presence of mPC by flow-MRD. **Indicated are**: Clinical response status (RS) at 1^st^ flow-RA and overall (best reached until DLC), relapse from flow-MRD negativity (flow-MRD status), sFLCR, SPEP and IF status, and corresponding time intervals between occasions (Δ). Biochemical abnormality (bioch. abn.) summarizes sFLCr, SPEP and IF (earliest timepoint is indicated). **Grey colour** highlights: Included patients, reaching RS og CR/SCR, progression, flow-MRD positivity or biochemical positivity. **Abbreviations:** **PID:** Patient ID. **PROG:** Clinical progression. **RS:** clinical response status. **PR:** Partial response. **VGPR:** Very good partial response. **CR:** Complete response. **SCR:** Stringent complete response. **SFLCr:** Serum free light chain ratio. **SPEP:** Serum protein electrophoresis. **IF:** serum immunofixation. **Neg:** negative. **Pos:** Positive. *****: Not determined/ not reached. **Abn:** Abnormality. **DLC:** Date of last contact or censoring date. **EXCL:** **<CR**: RS below CR, **NR:** BM sample not received for flow-MRD. **INCL:** Inclusion. **RA:** Response assessment. **FU**: Follow up flow-MRD sample.

**Table S4: Monitoring the quantity of flow-MRD until progression (6 patients)**

*
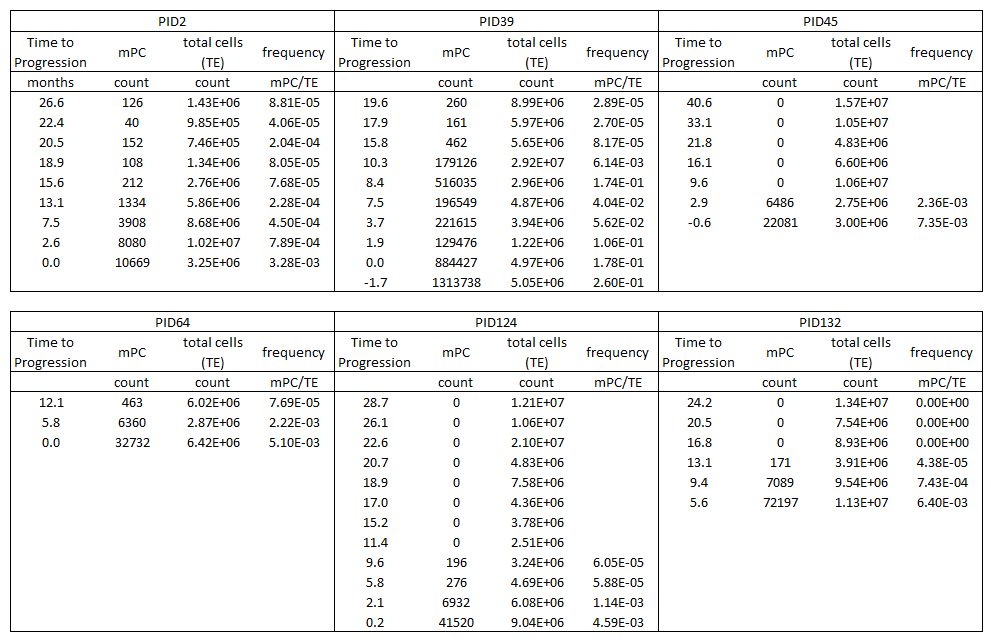
*

**Table S4 legend: Abbreviations: PID:** Patient ID. **TE:** Total acquired events (total cells). **mPC:** Abberrant plasma cells. **Count:** Absolute counts among acquired total cells.

**3  *Supplementary References (appear as references [28-34] in the main article)***

1. Kalina T, Flores-Montero J, van der Velden VHJ, Martin-Ayuso M, Böttcher S, Ritgen M, et al. EuroFlow standardization of flow cytometer instrument settings and immunophenotyping protocols. Leukemia [Internet]. 2012 Sep 5 [cited 2012 Nov 6];26(9):1986–2010. Available from: http://www.pubmedcentral.nih.gov/articlerender.fcgi?artid=3437409&tool=pmcentrez&rendertype=abstract

2. Hofste op Bruinink D, Oliva S, Rihova L, van der Holt B, Gilestro M, te Marvelde JG, et al. Flowcytometric Minimal Residual Disease Assessment in the EMN-02/HOVON-95 MM Trial: Used Methods and a Comparison of Their Sensitivity. Blood [Internet]. 2016 Dec 2;128(22):2072–2072. Available from: https://ash.confex.com/ash/2016/webprogram/start.html

3. Almeida J, Oreao A, Ocqueteau M, Mateo G, Corral M, Caballero MD, et al. High-sensitive immunophenotyping and DNA ploidy studies for the investigation of minimal residual disease in multiple myeloma. Br J Haematol. 1999;107(1):121–31.

4. Pérez-Persona E, Vidriales M-B, Mateo G, García-Sanz R, Mateos M-V, de Coca AG, et al. New criteria to identify risk of progression in monoclonal gammopathy of uncertain significance and smoldering multiple myeloma based on multiparameter flow cytometry analysis of bone marrow plasma cells. Blood [Internet]. 2007 Oct 1;110(7):2586–92. Available from: https://ashpublications.org/blood/article/110/7/2586/103625/New-criteria-to-identify-risk-of-progression-in

5. Jelinek T, Bezdekova R, Zatopkova M, Burgos L, Simicek M, Sevcikova T, et al. Current applications of multiparameter flow cytometry in plasma cell disorders. Blood Cancer J [Internet]. 2017 Oct 20;7(10):e617–e617. Available from: http://www.nature.com/articles/bcj201790

6. Arroz M, Came N, Lin P, Chen W, Yuan C, Lagoo A, et al. Consensus guidelines on plasma cell myeloma minimal residual disease analysis and reporting. Cytom Part B Clin Cytom [Internet]. 2016 Jan;90(1):31–9. Available from: http://www.ncbi.nlm.nih.gov/pubmed/25619868

# 
